# The Utility of Graph Clustering of 5S Ribosomal DNA Homoeologs in Plant Allopolyploids, Homoploid Hybrids, and Cryptic Introgressants

**DOI:** 10.3389/fpls.2020.00041

**Published:** 2020-02-10

**Authors:** Sònia Garcia, Jonathan F. Wendel, Natalia Borowska-Zuchowska, Malika Aïnouche, Alena Kuderova, Ales Kovarik

**Affiliations:** ^1^Institut Botànic de Barcelona (IBB, CSIC - Ajuntament de Barcelona), Barcelona, Spain; ^2^Department of Molecular Epigenetics, Institute of Biophysics, Academy of Sciences of the Czech Republic, Brno, Czechia; ^3^Department of Ecology, Evolution & Organismal Biology, Iowa State University, Ames, IA, United States; ^4^Faculty of Natural Sciences, Institute of Biology, Biotechnology and Environmental Protection, University of Silesia in Katowice, Katowice, Poland; ^5^UMR CNRS 6553 ECOBIO, Université de Rennes 1, Rennes, France

**Keywords:** 5S rRNA genes, allopolyploidy, hybridization, evolution, graph structure clustering, high-throughput sequencing, repeatome

## Abstract

**Introduction:**

Ribosomal DNA (rDNA) loci have been widely used for identification of allopolyploids and hybrids, although few of these studies employed high-throughput sequencing data. Here we use graph clustering implemented in the RepeatExplorer (RE) pipeline to analyze homoeologous 5S rDNA arrays at the genomic level searching for hybridogenic origin of species. Data were obtained from more than 80 plant species, including several well-defined allopolyploids and homoploid hybrids of different evolutionary ages and from widely dispersed taxonomic groups.

**Results:**

(i) Diploids show simple circular-shaped graphs of their 5S rDNA clusters. In contrast, most allopolyploids and other interspecific hybrids exhibit more complex graphs composed of two or more interconnected loops representing intergenic spacers (IGS). (ii) There was a relationship between graph complexity and locus numbers. (iii) The sequences and lengths of the 5S rDNA units reconstituted *in silico* from k-mers were congruent with those experimentally determined. (iv) Three-genomic comparative cluster analysis of reads from allopolyploids and progenitor diploids allowed identification of homoeologous 5S rRNA gene families even in relatively ancient (c. 1 Myr) *Gossypium* and *Brachypodium* allopolyploids which already exhibit uniparental partial loss of rDNA repeats. (v) Finally, species harboring introgressed genomes exhibit exceptionally complex graph structures.

**Conclusion:**

We found that the cluster graph shapes and graph parameters (k-mer coverage scores and connected component index) well-reflect the organization and intragenomic homogeneity of 5S rDNA repeats. We propose that the analysis of 5S rDNA cluster graphs computed by the RE pipeline together with the cytogenetic analysis might be a reliable approach for the determination of the hybrid or allopolyploid plant species parentage and may also be useful for detecting historical introgression events.

## Introduction

It is well-established that all modern plant species have experienced at least one whole genome duplication and that many also have interspecific hybridization and recurrent introgression in their recent history (Wendel, 2015; Alix et al., 2017; Nieto Feliner et al., 2017; Van De Peer et al., 2017). Documenting recent allopolyploidy is relatively straightforward using cytogenetic analysis and genome size measurements, since allopolyploids have twice as many chromosomes (or more) as the parental species. Identification of homoploid hybrids is more difficult since the chromosome number and genome size are often similar to that of the parental species (Nieto Feliner et al., 2017). Evolutionary young allopolyploids and other hybrids tend to retain fixed polymorphisms at protein-coding and non-coding loci. These duplicated loci are called homoeologs (Glover et al., 2016) and are useful for documenting parentage as well as understanding the dynamics of polyploid genomes (Yoo et al., 2014; Wendel, 2015; Bourke et al., 2018). Older allopolyploids can have experienced episodes of intergenomic translocation, dysploidy, gene conversion, localized deletions, and other genetic events, leading eventually to diploidization of the genome (Wendel, 2015; Wendel et al., 2018).

Ribosomal RNA genes encoding 5S, 5.8S, 18S, and 26S ribosomal RNA are ubiquitous in plants and are organized into arrays containing hundreds to thousands of tandem repeats at one or more genomic loci (Hemleben and Zentgraf, 1994; Nieto Feliner and Rosselló, 2007; Roa and Guerra, 2012; Garcia et al., 2017). Due to their rapidly diverging intergenic (IGS) and internally transcribed spacers (ITS), rDNA loci have become popular taxonomic markers revealing allopolyploidy and other interspecific hybridization in many plant and animal systems (Alvarez and Wendel, 2003; Poczai and Hyvonen, 2010; Nieto Feliner and Rossello, 2012). The internet searches using *ITS* and *allopolyploidy* resulted in more than 650 hits in Web of Science for just 2019. Most studies have used classical single clone sequencing approaches whereas high-throughput data have only rarely been employed and are limited to the 35S (45S) rDNA (Matyasek et al., 2012; West et al., 2014; Boutte et al., 2016). The analysis of 5S rDNA is also informative and has been successfully used in many phylogenetic studies (Cronn et al., 1996; Fulnecek et al., 2002; Baum et al., 2004; Besendorfer et al., 2005; Volkov et al., 2007; Baum et al., 2012; Jang et al., 2016). Its analysis is complementary to that of 35S since both loci usually occur separately on chromosomes (Roa and Guerra, 2012; Garcia et al., 2017). The 5S rDNA is usually located in one chromosome pair in most angiosperms and can occupy variable chromosome positions. It is organized in tandemly arranged units comprising hundreds to tens of thousands of copies. Each unit is composed of a conserved c. 120 bp coding region separated by a variable intergenic spacer (Sastri et al., 1992). Similar to 35S loci, 5S rDNA loci undergo concerted evolution, a process maintaining high homogeneity within and often between arrays (Dover, 1982; Elder and Turner, 1995; Parks et al., 2019). Such a process may rapidly homogenize rDNA sequences and induce copy number variation (Bughio and Maggert, 2019) blurring their hybridogenic signatures in allopolyploids (Wendel et al., 1995a; Volkov et al., 1999; Muir et al., 2001; Matyasek et al., 2003). In contrast to 35S rDNA, the 5S rDNA loci appear to be less sensitive to homogenization in some allopolyploids (Fulnecek et al., 2002; Pedrosa-Harand et al., 2006; Weiss-Schneeweiss et al., 2008; Garcia et al., 2017), retaining diagnostic capacity with respect to their parental origin.

The clustering algorithm employed by RepeatExplorer (RE) (Novak et al., 2010; Novak et al., 2013) has become a tool of choice for the analysis of chromosome composition and genome evolution (Renny-Byfield et al., 2012; Weiss-Schneeweiss et al., 2015; Ribeiro et al., 2017; Mlinarec et al., 2019; Peska et al., 2019). The phylogenetic signal of the repeatome has also been exploited in phylogenetic studies (Dodsworth et al., 2015; Dodsworth et al., 2016; Grover et al., 2019; Vitales et al., 2019). The analysis of genomes by RE is based on an all-to-all comparison of sequence reads revealing their similarities. Subsequently, the data are used to build clusters of overlapping reads representing different repetitive elements. The TAREAN tool, recently introduced into the RepeatExplorer2 pipeline, allows repeat identification and reconstruction of tandem repeats solely from sequence reads (Novak et al., 2017). Graph theory and connected component methods lying in the heart of the computation algorithm produce graph structures reflecting genomic organization of repeats. Typically, tandem repeats exhibit circular (ring) shape topologies are characterized by high values of circularity parameters. Although the RepeatExplorer2/TAREAN tool was initially developed for identification of non-coding satellites, 5S rDNA can also be analyzed with the program. This is because 5S rDNA shows many features of satellite repeats: (I) its highly homogeneous units are tandemly arranged in a head to tail orientation, (II) it appears in high copy number, allowing analyses even at low coverage, and (III) the size of 5S rDNA monomers (c. 200–1,000 bp) (Sastri et al., 1992; Fulnecek et al., 2006) falls within the range defined for satellite DNA, allowing circularization of chains of overlapping reads.

In this study we investigated the 5S rDNA genomic organization and homogeneity in more than 80 plant diploids and polyploids, exploiting high-throughput reads available from read archives in public genomic databases and also *de novo* sequenced by us. Particular attention was paid on hybrid systems with well-defined evolutionary histories, both eudicots and monocots: (i) *Brachypodium hybridum* (Poaceae), *Brassica carinata* (Ethiopian mustard, Brassicaceae), *Chenopodium quinoa* (quinoa, Amaranthaceae), *Gossypium hirsutum* (cotton, Malvaceae), and *Nicotiana rustica* (Aztec tobacco, Solanaceae) allotetraploids. (ii) *Spartina* × *townsendii* (cordgrass, Poaceae) homoploid hybrid. (iii) Species with frequent introgression events included *Gossypium gossypioides* and *Thinopyrum intermedium* (intermediate wheatgrass, Poaceae). We used the RepeatExplorer2/TAREAN clustering pipeline and cluster graph computation methods to address the following questions: (1) What is the relationship between graph complexity and intragenomic heterogeneity of 5S rDNA repeats? (2) Can the full-length 5S rDNA units be assembled from short sequence reads? (3) Can allopolyploids and other interspecific hybrids be distinguished from their progenitors based on cluster graph topologies? We show that cluster graphs may represent a convenient and simple-use approach for identification of interspecific hybrids from high-throughput sequencing data.

## Results

### Relationship Between Cluster Graph Topology and Intragenomic Diversity of 5S rDNA

**Figure 1:**
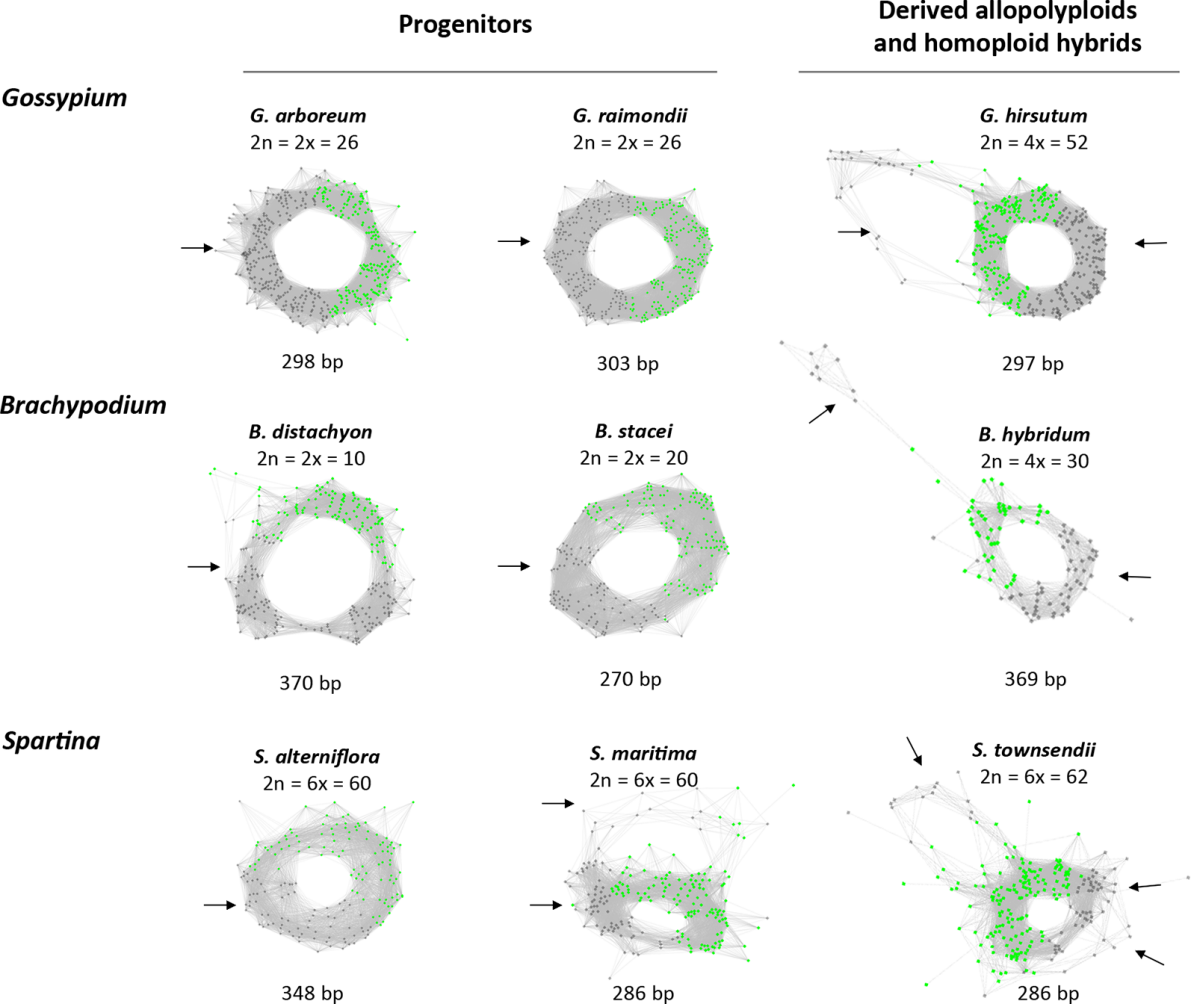
The 5S rDNA sequence reads organized in graph structures from the RepeatExplorer2 graphical output. Single reads are represented by vertices (nodes) and their sequence overlaps by edges. The 5S coding sequences and intergenic spacers are highlighted in green and grey vertices, respectively. Note, regular circular structures (referred to type 1) in most progenitors, and complex structures (referred to type 2) in derived allopolyploid and homoploid hybrids. Arrows indicate one or several intergenic spacers.

All of the 5S RNA gene families analyzed share a conserved c. 120 bp coding region while they differ in their intergenic spacers. We tested the hypothesis that the graph topologies of 5S rDNA clusters reflect the divergence and number of homoeologous gene families in allopolyploid genomes. Under this hypothesis, diploid species with a single gene family (and a locus) would generate a simple circular graph while allopolyploid and other hybrid genomes with multiple gene families (and loci) would display more complex graphs. To test this hypothesis we examined 5S rDNA cluster graph topologies in 84 plant species (**Supplementary Table S1**). Examples of cluster graph analyses in *Gossypium, Brachypodium*, and *Spartina* hybrid systems are shown in **Figure 1**. We have chosen these species because the parental genome donors, number of 5S rDNA loci, and approximate ages are known (**Table 1**). In the graphs, each vertex represents a sequence read and nodes connecting vertices depict sequence similarity between the reads. Simple circular 5S graphs with no or little deviation from regular circularity (referred as type 1 graphs) were observed in *G. arboreum, G. raimondii, B. distachyon*, and *B. stacei* diploid species. Except for the hexaploid *S. alterniflora*, the 5S graph topologies were more complex in polyploids (**Figure 1**). Specifically, two or more loops (rings) interconnected by a junction region (composed of 5S coding sequences) could be recognized. These complex structures are referred as type 2 graphs. Both loops were composed of vertices depicted in grey in **Figure 1** representing variable IGS regions. The total *k-mer coverage* scores (mean of cluster homogeneity) were high in diploid species while they were lower in the allotetraploids (**Table 1**). The *connected component index* C (mean of graph circularity) was uniformly high across the species. The read richness varied between the loops. For example, in *Brachypodium hybridum*, the right loop contained far more reads than the left loop.

**Table 1:** Cytogenetic characteristics of 5S rDNA loci and cluster graph parameters in allopolyploid and homoploid species and their progenitors.

	Ploidy level	N. loci/1C	N. reads in the cluster	Genome proportion (%)	Repeat size (bp)	k-mer coverage	Connected component index C	Graph shape (type)
*G. hirsutum*^1^	4x	2	351	0.170	297	0.660	0.997	2
*G. arboreum*	2x	1	418	0.210	303	1.000	1.000	1
*G. raimondii*	2x	1	386	0.190	298	0.974	0.974	1
*B. hybridum*^2^	4x	2	109	0.054	303	0.680	0.982	2
*B. distachyon*	2x	1	266	0.130	370	0.810	0.981	1
*B. stacei*	2x	1	239	0.120	270	0.950	1.000	1
*S*. × *townsendii*^3^	6x	n.d.	225	0.044	286	0.593	0.947	2
*S. alterniflora*	6x	n.d.	123	0.031	348	0.871	0.976	1
*S. maritima*	6x	n.d.	210	0.053	286	0.628	0.943	2

^1^G. hirsutum (2n = 4x = 52, AADD genome composition) is thought to originate from hybridization of species similar to modern G. raimondii (2n = 2x = 26, D genome donor) and G. arboreum (2n = 2x = 26, A genome donor).

^2^B. hybridum (2n = 4x = 30) is a natural allotetraploid with divergent subgenomes derived from diploid species similar to modern B. distachyon (2n = 2x = 10) and B. stacei (2n = 2x = 20).

^3^S. × townsendii (2n = 6x = 62) is a natural homoploid hybrid derived from S. alterniflora (2n = 6x = 62) and S. maritima (2n = 6x = 60).

**Figure 2:**
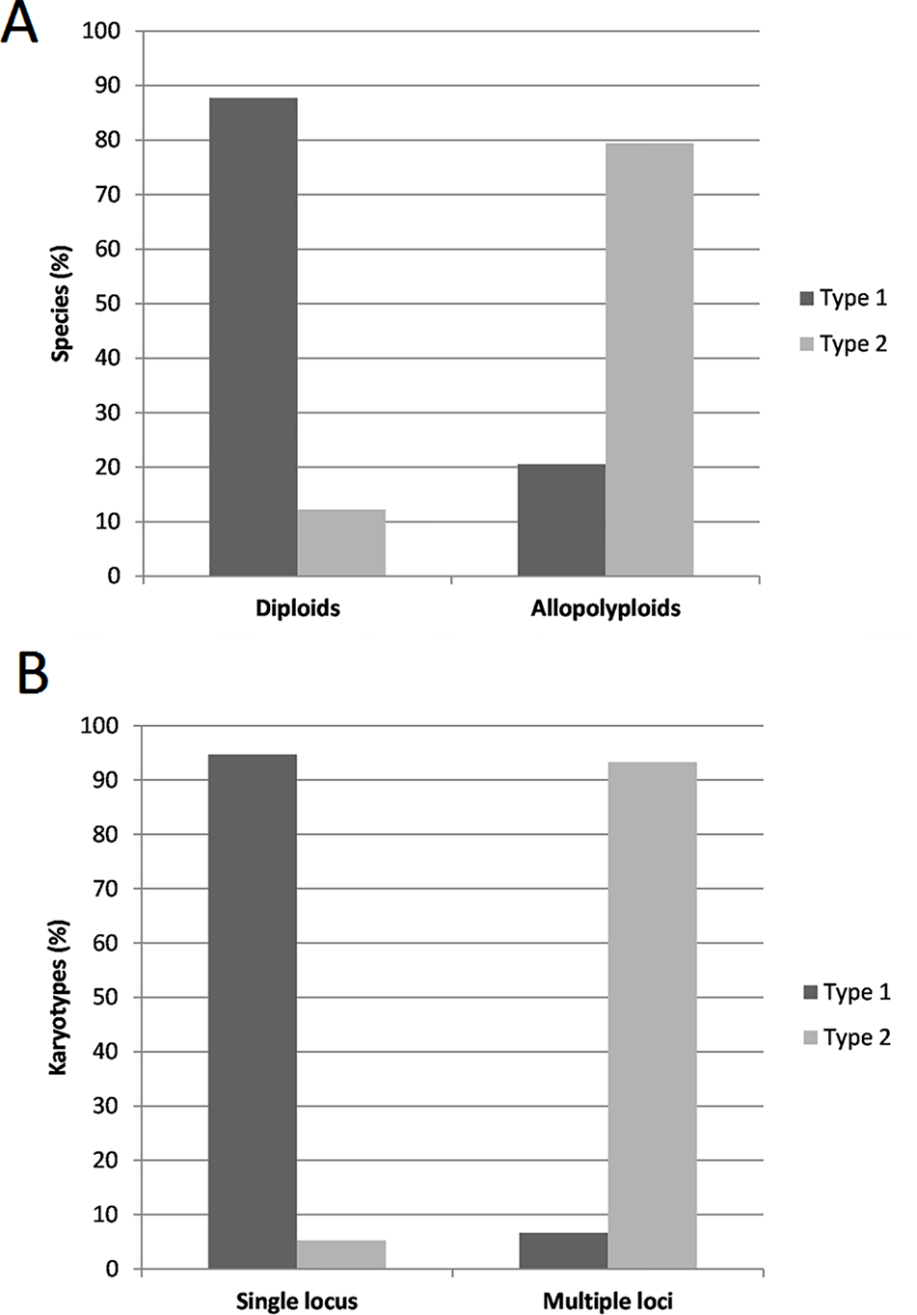
**(A)** Distribution of 5S rDNA type 1 and type 2 cluster graph structures between diploid (N = 50) and allopolyploid (including homoploid hybrids) (N = 32) species from the 84 species data set in this study. **(B)** Occurrence of type 1 and type 2 cluster graph structures in karyotypes with single (N = 38) and multiple (N = 30) 5S rDNA loci (data on 5S rDNA loci number only available for 68 species analyzed). The source data and the basic statistics are in **Supplementary Table S2**.

In the whole dataset (**Supplementary Table S1**) typical circular graph shapes of 5S rDNA clusters (type 1) were obtained in 81 (96%) species. The *connected component index* C parameter values (reported by a TAREAN) were high, ranging from 0.684 to 1.00 (average 0.959, s.d. 0.0599). In three species (4%), no circularization of 5S graphs clusters was obtained. **Figure 2A** shows the frequency of individual cluster types in allopolyploid and diploid species. The majority (87%) of diploid species showed type 1 structures while most (79%) allopolyploids displayed type 2 graphs (one-way ANOVA, F = 75.507, p < 0.001, **Supplementary Table S2**). About 95% single locus karyotypes displayed type 1 graphs while most (94%) karyotypes with two or more loci had type 2 structures (**Figure 2B**). There was a relationship between locus number and graph complexity (one-way ANOVA, F = 24.259, p < 0.001) with type 1 structures showing significantly lower locus numbers. The sequence homogeneity within each 5S rDNA cluster was estimated based on *total k-mer coverage* score reported by TAREAN and ranged 0.416–1.00 (average 0.760, s.d. 0.1392). The k-mer coverage values were significantly higher (one-way ANOVA, F = 200.363, p < 0.001, **Supplementary Table S2**) in the diploids compared to the allotetraploids and other hybrids (**Supplementary Figure S1**).

### Tracking the Origin of 5S rDNA Families in *Gossypium*, *Brachypodium*, and *Spartina* Allopolyploids and Homoploid Hybrids by Comparative Cluster Analysis

**Figure 3:**
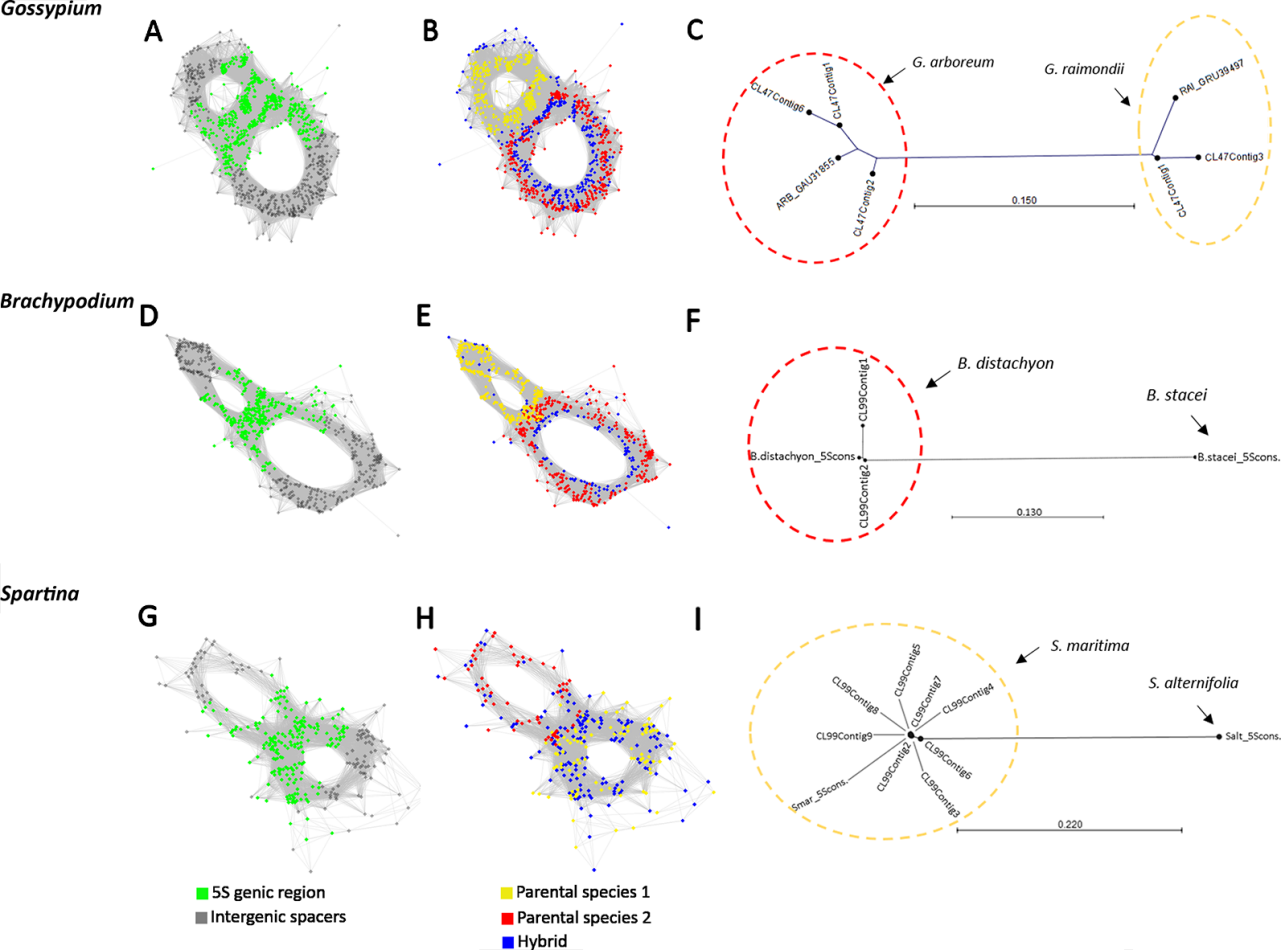
RepeatExplorer2 graphical output of the three-genomic comparative 5S rDNA analyses involving progenitor species and derived hybrids. **(A, D, G)** Graphs with highlighted 5S rDNA genic regions in green. **(B, E, H)** Cluster graphs with annotated reads origin: yellow vertices represent reads of one of the parental species (*G. raimondii* in **B**, *B. stacei* in **E**, and *S. maritima* in **H**); red vertices represent reads of the other putative parental species (*G. arboreum* in **B**, *B. distachyon* in **E**, and *S. alterniflora* in **H**); blue vertices represent reads from the hybrids and allotetraploids (*G. hirsutum* in **B**, *B. hybridum* in **E**, and *S*. × *townsendii* in **H**). **(C, F, I)** Neighbor joining phylogenetic trees constructed from aligned contigs from hybrids and progenitor 5S rDNA sequences.

Next, we investigated whether the homoeologous 5S genes can be visualized in cluster graph layouts and whether homoeologous gene families occur in assembled contigs. To address these questions we carried out a comparative three-genomic analysis (**Figure 3**) where inputs for clustering include reads from hybrids (allopolyploids) and their putative progenitor species. The overall cluster shapes were similar as in single genome analyses (**Figure 1**) indicating that the progenitor 5S rDNA sequences overlap with those of the derived hybrids and allopolyploids confirming, thus, their putative origin. Reads derived from 5S coding sequences (in green) were found in the junction region connecting both loops (**Figures 3A, D, G**). In **Figures 3B, E, H** reads originating from each progenitor (red and yellow) and hybrid (blue) genomes are labeled by different colors showing the 5S rDNA variants origin:

1. **Figure 3B** shows three-genomic cluster graph structures in *Gossypium hirsutum* and its progenitors. *G. hirsutum* is a 1–2 M years-old allotetraploid composed of subgenomes close to *G. arboreum* (A genome species) and *G. raimondii* (D genome species) (Wendel, 1989). The blue colored reads from the *G. hirsutum* allopolyploid were found in all parts of the graph—both in the junction region and loops; the red color reads from the *G. arboreum* diploid progenitor were located in the right loop and the junction region; the yellow colored reads originating from the other parental species, G. *raimondii*, were located in the left loop and the junction region. Similar cluster graph shapes were observed in remaining four *Gossypium* allotetraploids (*G. barbandense*, G. *mustelinum*, *G. darwinii*, and *G. tomentosum*, see **Supplementary Figure S2**), all having a similar AADD composition of the genome. In order to determine the identity of loop structures in the graphs we carried out a phylogenetic analysis of assembled 5S rDNA contigs (**Figure 3C** and **Supplementary Figure S2**). On the trees, sequences of both progenitors were well resolved forming separate branches, consistent with sequence divergence. The contigs from the *G. hirsutum* cluster grouped within the *G. arboreum* and *G. raimondii* branches, respectively.
2. **Figure 3E** shows three-genomic cluster graph structures in *Brachypodium hybridum* and its progenitors. *B. hybridum* is a c. 1 M years-old allotetraploid composed of subgenomes close to *B. distachyon* and *B. stacei* (Catalán et al., 2012). The comparative cluster graph displayed two loops composed of reads either from the *B. distachyon* (in red) or *B. stacei* (yellow). In contrast, reads from *B. hybridum* (blue) were shared between both loops. However, there were much less *B. hybridum* reads in the *B. stacei* loop compared to that of the *B*. *distachyon*. No B. *stacei* homoeologs were found among the contigs (**Figure 3F**).
3. **Figure 3H** shows three-genomic cluster graph structures in *Spartina* × *townsendii* and its progenitors*. Spartina* × *townsendii* is a less than 150 years-old homoploid hybrid composed of subgenomes inherited from *S. alterniflora* and *S. martima* hexaploids (Ainouche et al., 2004). The bottom read-rich circle contained sequences from the *S. maritima* (yellow) parent and *S*. × *townsendii* (blue); the upper read-poor circle was mostly formed by reads from *S. alterniflora* (red) parent and a few reads from *S*. × *townsendii* (blue). The junction region contained 5S genic sequences plus part of the IGS (grey) indicating short conserved sequences flanking the genic region. Another region of homoeologous genes similarity seems to exist in the middle of IGS indicated by interconnected reads from all three genomes (arrow). On the tree (**Figure 3I**), sequences from both progenitors were well resolved forming separate branches. However, all the assembled contigs grouped exclusively with the *S. maritima* branch.

Additional examples of 5S rDNA cluster analyses are shown in the **Supplementary Figure S3** comprising the well-known allopolyploids, *Brassica carinata* (4x), *Chenopodium quinoa* (4x), and *Nicotiana rustica* (4x). All these species harbored complex type 2 graphs, in which at least one (*Chenopodium*) or both progenitors (*Brassica* and *Nicotiana*) could be identified.

### Comparative Analysis Reveals Genetic Complexity in Species With Cryptic Introgression Histories

**Figure 4:**
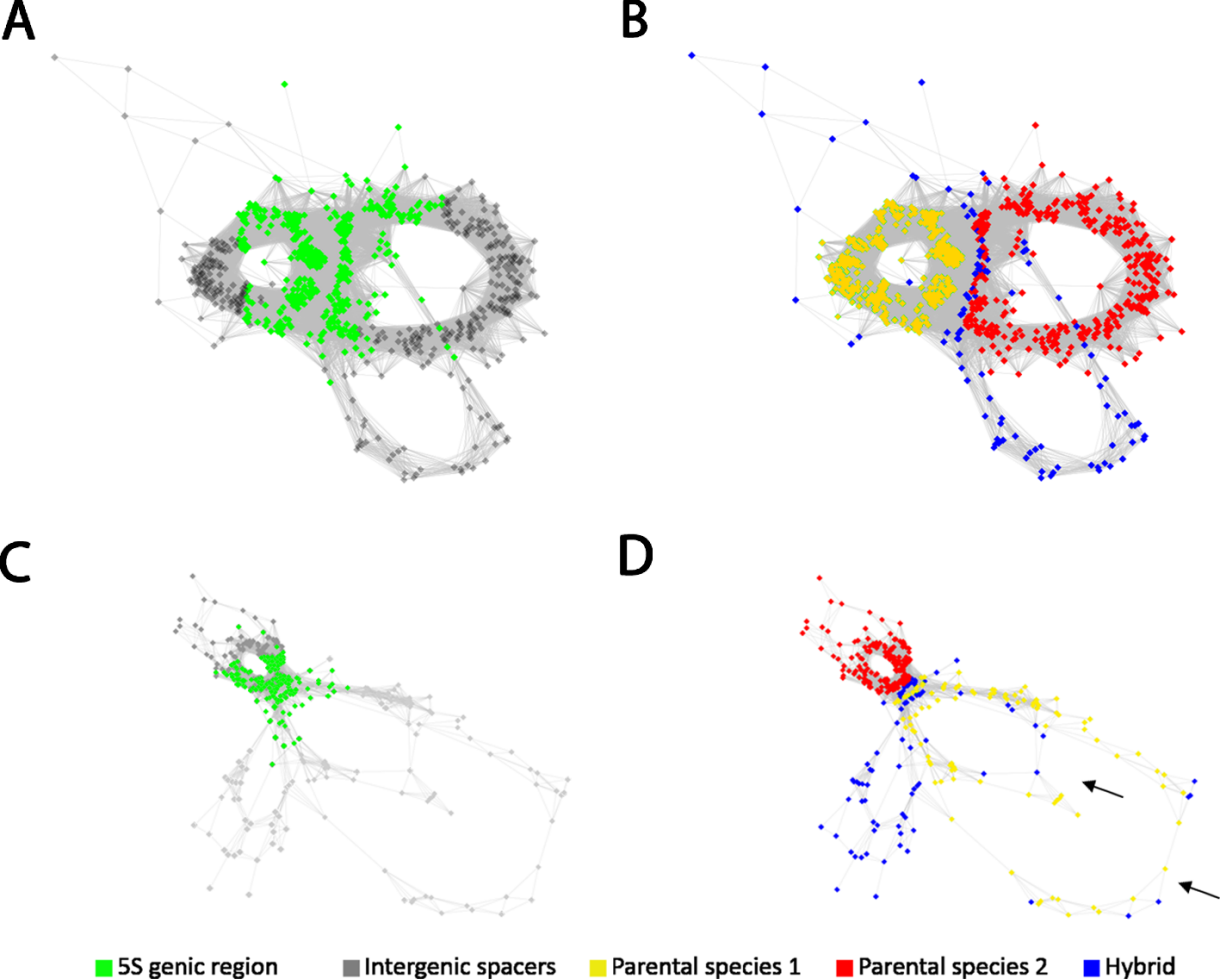
RepeatExplorer2 graphical output of the comparative analysis of the 5S rDNA clustering in species of hybrid origin and with complex evolutionary histories, in which introgressive hybridization may have been involved. Cluster graphs with highlighted 5S rDNA genic region in green **(A, C)** and annotated reads origin in **(B, D)** in *Gossypium gossypioides* and *Thinopyrum intermedium*, respectively. The comparative analysis in **(B)** mixing reads of the putative parental genome donors, *G. arboreum* as the **A** genome donor (red), *G. raimondii* as the **D** genome donor (yellow), and *G. gossypioides* as the hybrid (blue) shows that only few reads of the putative hybrid are placed in the **A** or **D** genome loops. The comparative analysis in **(D)** shows that the reads corresponding to the hexaploid *Thinopyrum intermedium* (blue) are partially shared with the reads of one of the putative parental genome donors, *Aegilops tauschii* (yellow) while there are no coincidences with the reads of the other putative parental genome donor, *Hordeum vulgare* (red). Arrows indicate two spacers of different sizes stemming from the *Aegilops* parental genome donor.

1. *Gossypium gossypioides* is a new world (D-genome) diploid species known to have experienced several rounds of introgressive hybridization from old world species (A genome) (Cronn et al., 2003). Its comparative cluster 5S rDNA graph of the three *Gossypium* species analyzed (**Figures 4A, B**) showed three loops where the *G. gossipioides* reads formed a unique loop (blue) that did not overlap with either A (red) or D (yellow) genome loops. Except for the genic junction region no significant interconnecting edges between the three genomes were visualized.
2. The intermediate wheatgrass *Thinopyrum intermedium* (Poaceae) is a hexaploid species experiencing multiple introgression events, potentially including genome parts from several species. We therefore included candidate *Aegilops tauschii* and *Hordeum vulgare* progenitor species in our comparative analysis of 5S rDNA (**Figures 4C, D**) which had been suggested as potential genome contributors for *T. intermedium* (Tang et al., 2000; Mahelka et al., 2011). At least four loops could be recognized on the cluster graph. Two loops contained shared reads from *T. intermedium* (blue) and *A. tauschii* (yellow). In addition, there was a prominent read-rich *Thinopyrum*-specific loop (**Figure 4D**, all-blue loop) that did not contain reads from other genomes and may originate from *Dasypirium* (Mahelka et al., 2013) for which read archives were unavailable. No *T. intermedium* reads were present in the *H. vulgare* loop (red).

### Quantification of 5S and 35S rDNA Homoeologs in *Gossypium* and *Brachypodium* Allotetraploids From High-Throughput Sequencing Data

In the cluster graphs of the hybrid species (**Figures 1** and **3**) we often observed differences in read-richness between both loops suggesting a skewed representation of homoeologous 5S rDNA variants. To validate this assumption we quantified homoeologous 5S rDNAs by mapping of Illumina reads to the reference sequences of 5S rDNA units (**Supplementary Figure S4**). In five *Gossypium* allopolyploids analyzed (**Supplementary Figure S4A**), the 5S rDNA homoeologs were slightly skewed toward the A genome units. This clearly contrasts with 35S rDNA (**Supplementary Figure S4B**), where all but one allotetraploids contained far fewer A-genome than D-genome ITS1 types, except of *Gossypium mustelinum* in which the homoelogous ratio was inversed. A similar analysis carried out in *Brachypodium hybridum* showed a higher representation of *B. distachyon* homoeologs (in this case, both 35S and 5S rDNA) than those of B. *stacei* (**Supplementary Figure S4C**).

### Reconstitution of 5S rDNA Units and Gene Copy Number in *Gossypium* Allotetraploids

The unit length is an important characteristic of rDNA arrays. We compared the lengths of *in silico* assembled *Gossypium* units with those previously determined by conventional cloning and Sanger sequencing (**Table 2**). RepeatExplorer2/TAREAN generates consensus sequences of 5S rDNA units from the decomposition of read sequences into k-mers (Novak et al., 2017). The lengths of 5S rDNA units determined by cloning ranged from 295–303 bp while those calculated from sequence data by bioinformatics tools ranged from 265–303 bp. In general, there was congruence between both methods. In contrast, the copy number variation between species was extremely high (up to 10 fold), confirming previous findings (Cronn et al., 1996). In some cases, copy numbers determined by computation methods differed by more than five-fold from those of slot blot hybridization experiments (Cronn et al., 1996).

**Table 2:** 5S rDNA unit lengths and copy number in *Gossypium* allotetraploids and diploids.

	SRA Identification	Unit length^1^		Copy number^2^	
		High-throughput data^4^	Cloning	High-throughput data^5^	Slot blot hybridization
*Gossypium mustelinum*	SRR769542	265	301–303	14,015	21,845
*Gossypium hirsutum^3^*	SRR768357	297	295–279	18,412	11,190
	ERR1449079	265	295–279	14,903	11,190
*Gossypium barbadense*	SRR8624709	265	296–298	18,157	23,515
*Gosspium raimondii*	ERR1449077	303	301–303	11,061	4,730
*Gossypium arboreum*	SRR1216970	298	297–298	23,691	7,550
*Gossypium thurberi*	SRR8076131	302	301–302	10,607	2,070
*Gossypium darwinii*	SRX5347640	273	n.d.^6^	24,276	n.d.
*Gossypium tomentosum*	SRR8815512	259	296–297	38,691	22,290
*Gossypium gossypioidies*	SRR8136267	297	301–303	3,292	1,145
*Gossypium herbaceum*	SRR617255	265	297–298	7,819	3,415
*Gossypium davidsonii*	SRR8136261	302	301–303	19,909	10,280

^1^Genic and intergenic region (bp). Data from sequencing of clones are from Cronn et al. (1996).

^2^Copy number in the somatic cell genome (2C). Slot blot hybridization results are from Cronn et al. (1996).

^3^Data are from two different accessions.

^4^K-mer assembly.

^5^Calculated from the genome proportion.

^6^n.d.—not determined.

### Southern Blot Hybridization Analysis of 5S rDNA in *Spartina*

**Figure 5:**
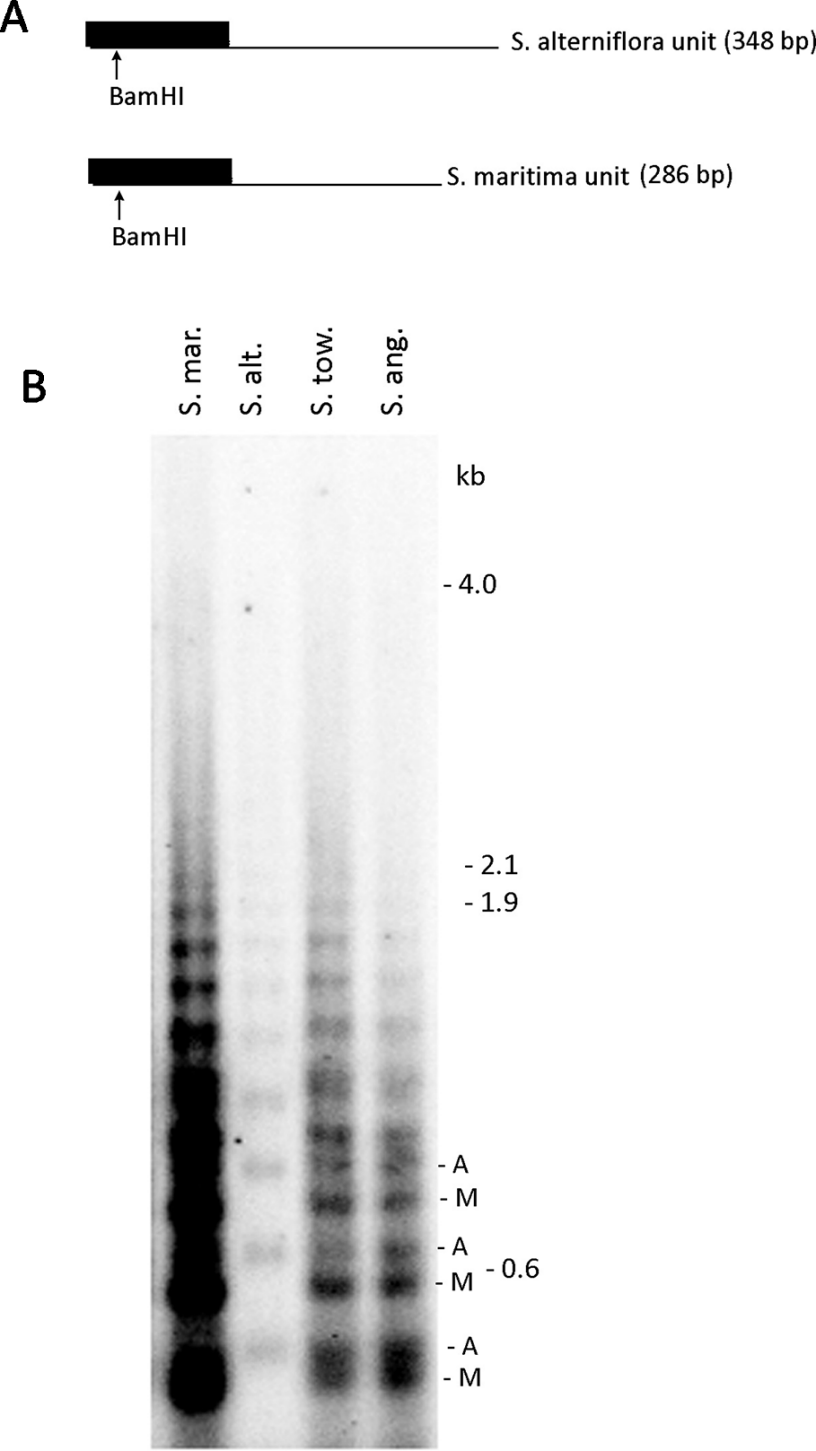
Southern blot analysis of 5S rDNA in *Spartina* hybrids and their progenitors. **(A)** Restriction maps of progenitor *S. maritima* and *S. alterniflora* of 5S rDNA. **(B)** The 5S rDNA probe hybridization to *S. maritima* (S. mar.), *S*. *alterniflora* (S. alt.), *S*. × *townsendii* homoploid hybrid (S. tow.), and *S. anglica* dodecaploid (S. ang.) DNAs. In *S*. × *townsendii* and *S. anglica*, hybridization fragments inherited from progenitors are marked as “A” (from *S. alterniflora*) and “M” (from *S. maritima*), respectively.

Previous *in silico* analyses showed highly skewed 5S rDNA homoeologs in *Spartina* × *townsendii* hexaploid toward the *S. maritima* genome. In order to confirm this result, we carried out southern blot hybridization using genomic DNA from *S*. × *townsendii* (*6x*), the derived *S. anglica* allododecaploid (12x), and the progenitors of both species, *S. maritima* and *S. alterniflora* (**Figure 5**). Genomic DNA was digested with *Bam*HI which has a conserved site in the angiosperm 5S rDNA units (Röser et al., 2001) (**Figure 5A**). The 5S rDNA probe generated ladders of bands, expected from a tandemly arranged sequence as the 5S rRNA genes. The probe hybridized strongly to the *S. maritima* DNA while the hybridization to *S. alterniflora* was relatively weak (**Figure 5B**). The *S. maritima* oligomers were slightly shorter than those of *S. alterniflora* consistent with shorter length of the *S. maritima* units (**Figure 1**). In both *S*. × *townsendii* and *S. anglica* oligomeric bands derived from both parents were visible indicating additivity.

## Discussion

Here we studied the organization and evolution of 5S rDNA in 84 plant genomes. We found that the clustering analysis of high-throughput Illumina reads by RepeatExplorer2/TAREAN provides a comprehensive view about 5S rDNA origin and organization, corroborating classical cytogenetic and molecular studies. The 5S rDNA cluster graphs are typically circular, showing high values of circularity parameters underlining regular tandem arrangement of these genes. Graphs displayed no or little node irregularities (discussed further below) consistent with a high homogeneity of arrays and confirming the model of concerted evolution (Dover, 1982; Kellogg and Appels, 1995). The sequence and size of *in silico* reconstituted 5S rDNA units were congruent with those obtained from cloning and Sanger sequencing. Below we discuss a value-added information obtained from cluster analyses that is not obtainable (or with difficulty) by conventional molecular and cytogenetic analyses.

### Dynamism of rDNA Loci in Hybrid Genomes

We investigated rDNAs in two relatively ancient (c.1 Myr) allopolyploid systems (*Gossypium* and *Brachypodium*) which already show substantial loss of 35S rDNA homoeologous units (Wendel et al., 1995a; Borowska-Zuchowska and Hasterok, 2017). *Gossypium* allotetraploids were represented by five species with a typical AADD genome composition originating from common diploid ancestors closely related to *G. arboreum* (A genome) and *G. raimondii* (D genome). In these allotetraploids, previous cloning analyses identified both homoeologous 5S rDNA sequences in *G. hirsutum* and *G. mustelinum* but not in *G. barbadense* and *G. tomentosum*, where only the A genome sequences were recovered (Cronn et al., 1996). However, comparative graph clustering of the 5S rDNA revealed both A and D genome homoeologs in these species, with a dominance of the A-genome units. Also, with respect to the 35S rDNA, the D-genome type of ITS in *G. mustelinum* sequence was barely detectable using Southern blot hybridization (Wendel et al., 1995a), but high-throughput sequencing recovers similar sequences at a frequency of about 15% (**Supplementary Figure S4B**). A similar example of skewed homoelog ratios is represented by *B*. *hybridum* and *S. × townsendii*, where one loop contained far more reads than the other in the 5S rDNA cluster graphs. Indeed, Southern blot analysis confirmed one strong and one weak 5S rDNA family in the *S*. × *townsendii* homoploid hybrid and *S. anglica*. Skewed gene ratios exist already in the progenitor genomes based on read abundance in the graph loops and differential intensity of Southern hybridization signals. A strong repeat-rich locus likely occurs in the *S. maritima* parent while a weaker locus may be present in the *S. alterniflora* parent. These examples demonstrate a higher sensitivity of a cluster graph-based approach over the *de novo* assembly or PCR-cloning approaches, where various technical biases may occur (Lunerova et al., 2017).

### Evaluation of the Graph-Based Method for Identification of Allopolyploids and Hybrids

Diploid genomes show simple circular structures of 5S rDNA cluster graphs referred to type 1 (**Figure 2A**). In contrast, allopolyploids and homoploid hybrids display more complex graph structures (type 2) in which divergent gene families are visualized as distinct loops. In addition, there was a good correlation between cluster graph complexity and number of 5S rDNA loci (**Figure 2B**). These observations are consistent with a general view that most diploids carry a single 5S rDNA locus (and a single gene family) while allopolyploids tend to maintain multiple loci (and multiple 5S rDNA families) (**Table 1**) and (Roa and Guerra, 2015; Garcia et al., 2017). Thus, a simple visual inspection of the 5S rDNA cluster topology appears to be informative with respect to the putative hybridogenic origin of a species. Among the computation parameters, the k-mer coverage seems to reflect the intragenomic homogeneity—low k-mer scores associate with complex graph shapes and multiple gene families, while high k-mer scores associate with simple circular structures and single gene families. Thus, the k-mer coverage may be taken as a semi-quantitative parameter of 5S rDNA intragenomic homogeneity, although more studies are needed to validate the relationship.

**Figure 6:**
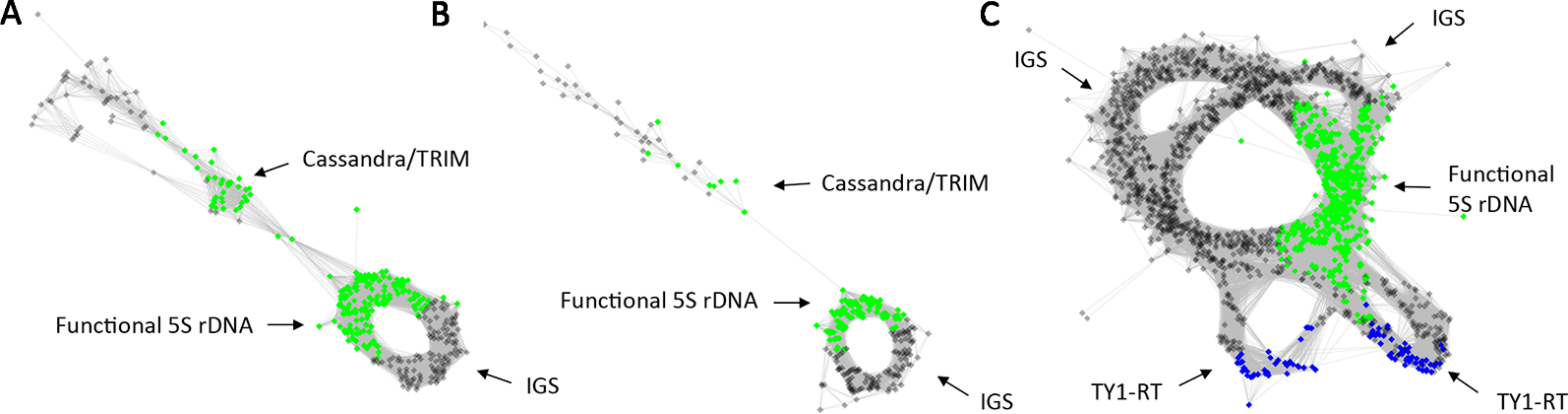
Graph structures of 5S rDNA clusters from the RepeatExplorer2 graphical output containing a significant number of retroelement sequences in *Tragopogon porrifolius* **(A)**, *Senecio vulgaris* **(B)** and *Musa acuminata* **(C)**. In **(A, B)** reads derived from a Cassandra/TRIM element. Note, the Cassandra/TRIM elements were relatively isolated and connected to 5S rDNA with 1–2 reads only. In **(C)** the highly complex structure of 5S rDNA in *M. acuminata* containing multiple IGS and regions of similarity to a reverse transcriptase domain of a TY-1 Copia retroelement.

One of the advantages of the clustering-based method is that it may provide initial information about the 5S rDNA homoeologs without prior knowledge of progenitor genomes based on the graph complexity. Certainly, the origin of 5S rDNA families in a hybrid is indicated by comparative clustering requiring sequences from candidate progenitor genomes. We observed similar graph complexities for the 35S rDNA encoding 18S-5.8S-26 rRNA genes (**Supplementary Figure S5**) suggesting that these clusters (particularly, 3' 26S region and the IGS) may be equally informative as that of 5S rDNA. These cases pose additional opportunities for studying the recombination dynamics of dispersed 5S and ITS arrays, which may be subject to complex and incomplete concerted evolutionary forces. Despite the apparent good correlation between cluster complexity and a hybrid character of the genome there were several notable exceptions from the rule:

1. About 21% of allopolyploids and homoploids showed simple type 1 graphs. These simple graphs can be explained by high similarity of progenitor units, preventing separation of reads. However, it can also be explained by locus loss and/or homogenization of 5S rDNA in allopolyploids over longer evolutionary times. Indeed, ancient (c.5 Myr) *Nicotiana* allotetraploids from section Repandae showed simple circular type 1 graphs (not shown) and a diploid character of 5S rDNA loci (Lim et al., 2007). Interestingly, *Triticum turgidum* (0.5 Myr) and *Spartina alterniflora* (3 Myr) (both Poaceae) polyploids also show simple cluster graphs despite their relatively young age, suggesting that the process of rDNA homogenization and diploidization may proceed at different rates in different systems. Frequent losses of 5S rDNA loci in *Triticum* (Baum et al., 2008), rice (Zhu et al., 2008) and *Spartina* (this work) polyploids may also suggest certain instability of 5S rDNA in Poaceae. Nevertheless, the *Thinopyrum* hybrid (Poaceae) displayed a highly complex cluster graph (**Figures 4C, D**) consistent with the retention of progenitor 5S rDNA families (Mahelka et al., 2013) and therefore arguing against generalization of these observations.
2. About 13% of diploids showed complex type 2 graphs, indicating intragenomic heterogeneity of 5S rDNA loci in these genomes. In at least some cases, the intragenomic heterogeneity of 5S rDNA in these diploids can be explained by homoploid hybridization and introgression events. This is probably the explanation for the complex graphs in *Gossypium gossypioides* (**Figure 4A**) which has a complex evolutionary history entailing at least two temporally widely separated divergence events (Wendel et al., 1995b; Cronn et al., 2003). Although introgression and hybridization is also relatively frequent in the banana genus (Němečková et al., 2018) a more likely explanation for complex graph structures in *Musa acuminata* (**Figure 6C**) is an exceptionally high number of 5S rDNA loci in this species (six per diploid genome) (Valarik et al., 2002; Garcia et al., 2012a) and probably inefficient interlocus recombination (Schlotterer and Tautz, 1994) leading to poor homogenization. Actually, the mechanisms of amplification of 5S rDNA loci across the chromosomes are still poorly understood (Schubert and Wobus, 1985; Symonova et al., 2017; Joachimiak et al., 2018; Souza et al., 2019).
3. The occurrence of non-rDNA sequences within the 5S rDNA clusters may potentially distort graph shapes. In *Tragopogon porrifolius* and *Senecio campestris* (both Asteraceae) the 5S rDNA clusters apparently contain traces of Cassandra transposable elements. These LTR elements are widespread in angiosperm genomes and carry a 5S rDNA related sequence (Kalendar et al., 2008). In cluster graphs the Cassandra element can be identified by divergent reads connected by only a few nodes to the 5S rDNA genic region (**Figures 6A, B**). The known high mobility of 5S rDNA in the *Musa* genus (Valarik et al., 2002) could be related to the activity of transposable elements whose remnants (TY1 copia/Tork family) are apparently found in some *M. acuminata* 5S rDNA units (**Figure 6C**). In general, the frequency of non-rDNA sequences was low (<4% analyses) in the major 5S rDNA clusters and likely does not represent significant source of artefacts.

### Concluding Remarks

To summarize, we infer that the visual inspection of rDNA cluster graph topologies coupled with calculation of graph parameters is highly informative for the assessment of rDNA genomic organization, number of rRNA gene families, and loci. The method may provide clues for testable hypotheses about evolutionary histories of interspecific hybrids and allopolyploids, especially in biological systems with unknown or not well defined genome donors (Mahelka et al., 2011; Kaplan et al., 2013; Fredotovic et al., 2014; Belyayev et al., 2018). It is necessary to stress that a robust evaluation of hybridization and polyploidy cannot be solely based on read clustering, but should involve a combination of various cytogenetic, molecular and genomic methods.

## Materials and Methods

### DNA Isolation, High-Throughput Sequencing, and Read Archive Accessions

Most sequences used in this study were downloaded from sequence read archives at the EBI server (**Supplementary Table S1**). Six genomes were sequenced *de novo* as follows: genomic DNA from leaf tissue was isolated by a modified CTAB method and sequenced by Illumina technology at BGI. The *Spartina* DNAs originated from natural samples collected in Southampton area, UK: *S. maritima* (Isle of Wight), *S*.× *townsendii* (Hythe), *S. alterniflora*, and *S. anglica* (both from Eling Marchwood) (Huska et al., 2016); *Cardamine × insueta* and *C. amara* were from natural populations in Urnerboden, Switzerland (Zozomova-Lihova et al., 2014); *C. flexuosa* was from Zelezne, Slovakia, and *C. hirsuta* from Gehausen, Germany (Mandakova et al., 2014). Details of sequencing are provided in **Supplementary Table S3**.

### *In Silico* Identification of 5S rDNA Repeats

The fastq reads were initially filtered for quality and trimmed to a uniform length by pre-processing and QC tools by RepeatExplorer2 (Novak et al., 2013). The pipeline is implemented in the Galaxy environment (https://galaxy-elixir.cerit-sc.cz/). For computation, resources at the international ELIXIR infrastructure (European research infrastructure for biological information) were used. Read length ranged 100–150 bp depending on sequencing library and platform. After the fastq > fasta conversion reads were analyzed with RepeatExplorer2 using default parameters. The RepeatExplorer2 pipeline runs a graph-based clustering algorithm (Novak et al., 2013) to assemble the groups of frequently overlapping reads into clusters of reads, representing a repetitive element, or part of a repetitive element with a higher order genome structure. It uses a BLAST threshold of 90% similarity across 55% of the read to identify reads to each cluster by default (minimum overlap = 55, cluster threshold = 0.01%, minimum overlap for assembly = 40), and the clusters are identified based on a principle of maximum modularity. Typically, 200,000 of pair-end reads were used as input for clustering. This number typically yields a cluster comprising several hundreds of 5S rDNA specific reads. Analysis of larger (>2 Gb/1C) genomes requires an increase of the number of input reads (up to 2 million), as the 5S rDNA coverage decreases. Although this prolongs computation times (typically 5–6 hours on the MetaCentrum ELIXIR computer clusters) we were able to reconstruct 5S rDNA units in the large (50 GB) *Fritillaria imperialis* genome (Zonneveld, 2010) although, in this case, the number of reads in the cluster was too low, preventing graph analysis. High coverages may also help to reveal rare 5S rDNA variants and pseudogenes that are frequent in gymnosperms (Wang et al., 2016; Wang et al., 2019) while they rarely occur in angiosperms. In interspecific comparisons, the usage of a standard fraction of genome (0.1–1.0%) is recommended to prevent biases in interspecific comparisons.

The 5S rDNA clusters were searched among the *cluster annotation* files using “rDNA” search keyword. Alternatively, 5S rDNA clusters were found in TAREAN tandem reports (a specific tool for the analysis of tandem repeats implemented in RepeatExplorer2). The shapes of cluster graphs were characterized by a *connected component index* parameter (C) which is calculated as the proportion of the largest strongly connected component in graph composed of oriented reads (Novak et al., 2017). Cluster graph topologies were visually inspected and categorized into two groups (simple, type 1, and complex, type 2, graphs). The k-mer score was calculated by the RepeatExplorer2/TAREAN program as the sum of frequencies of all k-mers used for consensus sequence reconstruction.

### Identification and Quantification of Homoeologous 5S rRNA Gene Families

5S rDNA homoeologous families were quantified by mapping analysis using CLC Genomics Workbench (QIAGEN), CLC onwards. Trimmed reads (typically >7 million) were mapped to the corresponding reference with following parameters: insertion and deletion costs = 3, lengths fraction = 0.5, similarity fraction = 0.9, deletion cost = 2. As reference sequences we used *Gossypium arboreum* (GenBank no. GAU31855) and *G. raimondii* (GRU39497) clones. Since no GenBank 5S rDNA clones were available for *Brachypodium* we used consensus sequences of *B. distachyon* (370 bp) and *B. stacei* (270 bp) generated by RepeatExplorer2 as a reference.

Phylogenetic analysis was carried out using assembled contigs computed by RepeatExplorer2. Briefly, BLAST libraries of contigs from hybrids and allopolyploids were BLASTed against the 5S rDNA sequences: For *Gossypium*, these were GenBank clones (#GAU31855 and #GRU39497); for *Brachypodium* and *Spartina* contigs generated by RepeatExplorer were used. The IGS subregions were extracted from BLAST outputs by a selection command in CLC, grouped and aligned. Alignments were manually edited and neighbor joining phylogeny trees constructed (CLC).

Because homoeologous ITS1 (internally transcribed spacer 1 of 35S rDNA) cannot be quantified by mapping procedures due to their overall similarity, we calculated the ITS1 homoeologous ratios from the number of nodes in genome-specific clades of phylogeny trees: (i) ITS sequences from *Gossypium* and *Brachypodium* allotetraploids were extracted from mapped reads, yielding typically hundreds to thousands of sequences. (ii) Stand-alone BLAST databases were generated from the ITS sequences. The databases were queried with reference sequences derived from variable 50–70 bp central subregions of ITS1. The ITS1 consensus sequences were obtained from the alignment of GenBank clones: *B. stacei* (JX665827-JX665832), *B. hybridum* (JX665718-JX665731), *G. arboreum* (GAU12712), and *G. raimondii* (GTU12711). (iii) Reads extracted from BLAST outputs were trimmed to uniform length, sampled (100–500 reads), and NJ trees constructed using a phylogeny tool of CLC. Homoeologous sequences in distinct clades were extracted, counted, and expressed as a ratio.

### Statistical Methods

We analyzed the data by one-way ANOVA statistical test implemented within the MS Office package (XL-Toolbox NG). Box-plots were constructed using an online BoxPlotR tool (www.shiny.chmgid.org/boxplotr/).

### Southern Blot Hybridization

Total genomic DNA was extracted from fresh young leaves using a modified CTAB method following procedures described previously (Kovarik et al., 1997). Genomic DNA was digested with the *Bam*HI restriction enzyme and hybridized on blots. The DNAs were digested with *Bam*HI and hybridized with the radioactively labeled ([32P] dCTP, Dekaprimer labeling kit (Thermo Fischer, USA) 5S rDNA probe. The probe was a trimer of the 5S rRNA gene from *Artemisia tridentata* [S4 clone, GenBank # JX101915.1, (Garcia et al., 2012b)]. Hybridization signals were visualized using a PhosphorImager (Fuji, FLA 9000).

## Data Availability Statement

The datasets generated for this study can be found in the GenBank Sequence Read Archives (Bioprojects “Chromosome evolution in invasive *Spartina* plants”, ID: PRJNA575642 and “Chromosome evolution in *Cardamine* hybrids and polyploids”, ID: PRJNA575831).

## Author Contributions

Conceived and designed the study: AKo, SG. Performed the experiments and collected material: SG, AKo, and MA. Analyzed the data: SG, MA, JW, NB-Z, AKu, and AKo. Wrote the manuscript: AKo, SG.

## Funding

The work was supported by the Czech Science Foundation (grant 19-03442S), the Polish OPUS project of the National Science Centre (2018/31/B/NZ3/01761) and by the Dirección General de Investigación Científica y Técnica (CGL2016-75694-P AEI/FEDER, UE) from the government of Spain. SG benefited from a Ramón y Cajal contract (RYC-2014-16608) from the government of Spain.

## Conflict of Interest

The authors declare that the research was conducted in the absence of any commercial or financial relationships that could be constructed as a potential conflict of interest.
