# Supplementary figures and images for: The Utility of Graph Clustering of 5S Ribosomal DNA Homoeologs in Plant Allopolyploids, Homoploid Hybrids, and Cryptic Introgressants
Source: Front Plant Sci. 2020 Feb 10;11:41. doi: 10.3389/fpls.2020.00041 (PMC7025596; doi:10.3389/fpls.2020.00041)

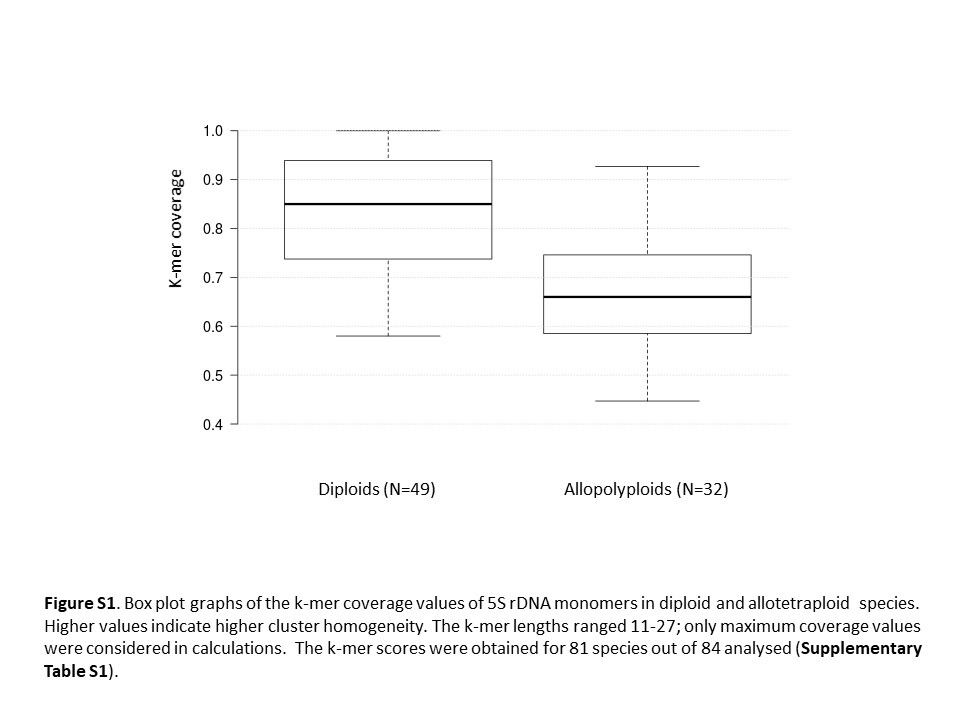

Supplement: Supplementary file 1 [file Image_1.jpeg]

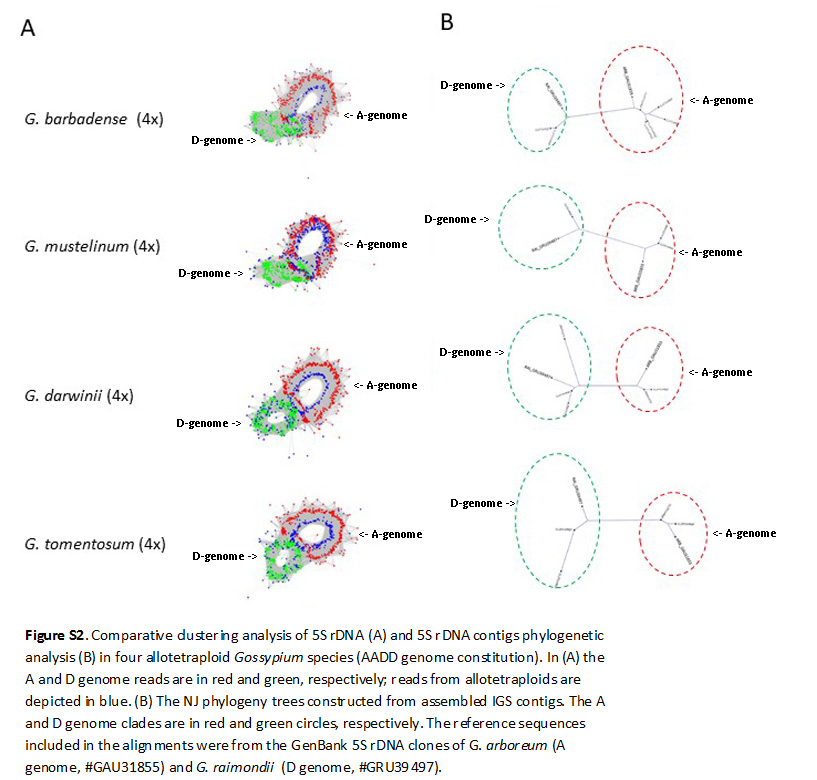

Supplement: Supplementary file 2 [file Image_2.jpeg]

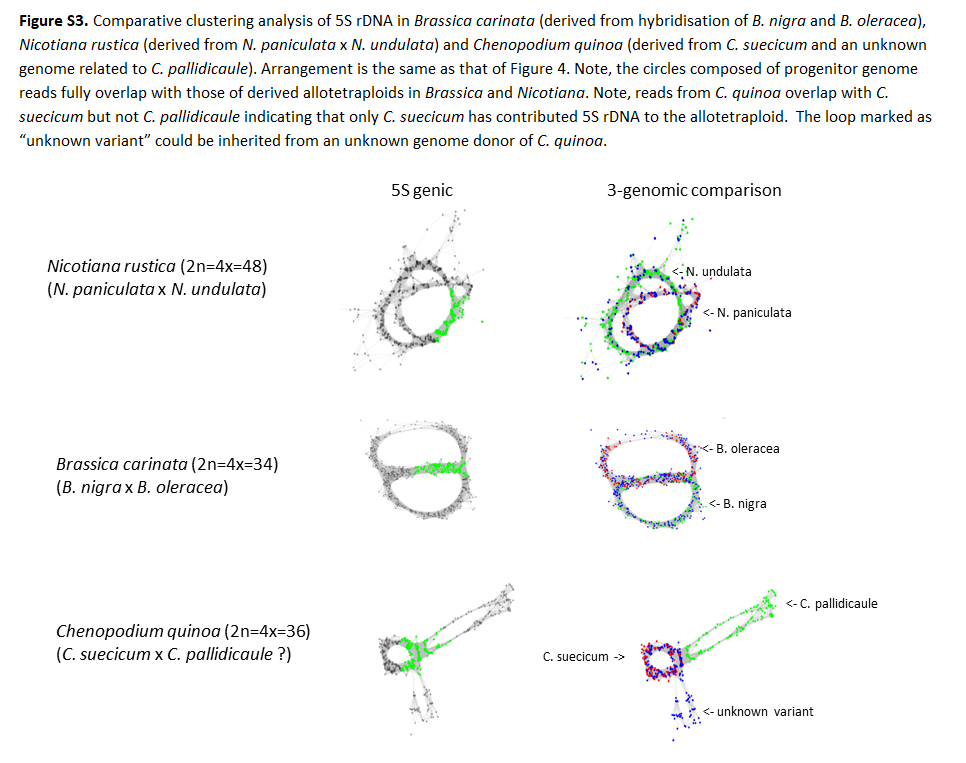

Supplement: Supplementary file 3 [file Image_3.tif]

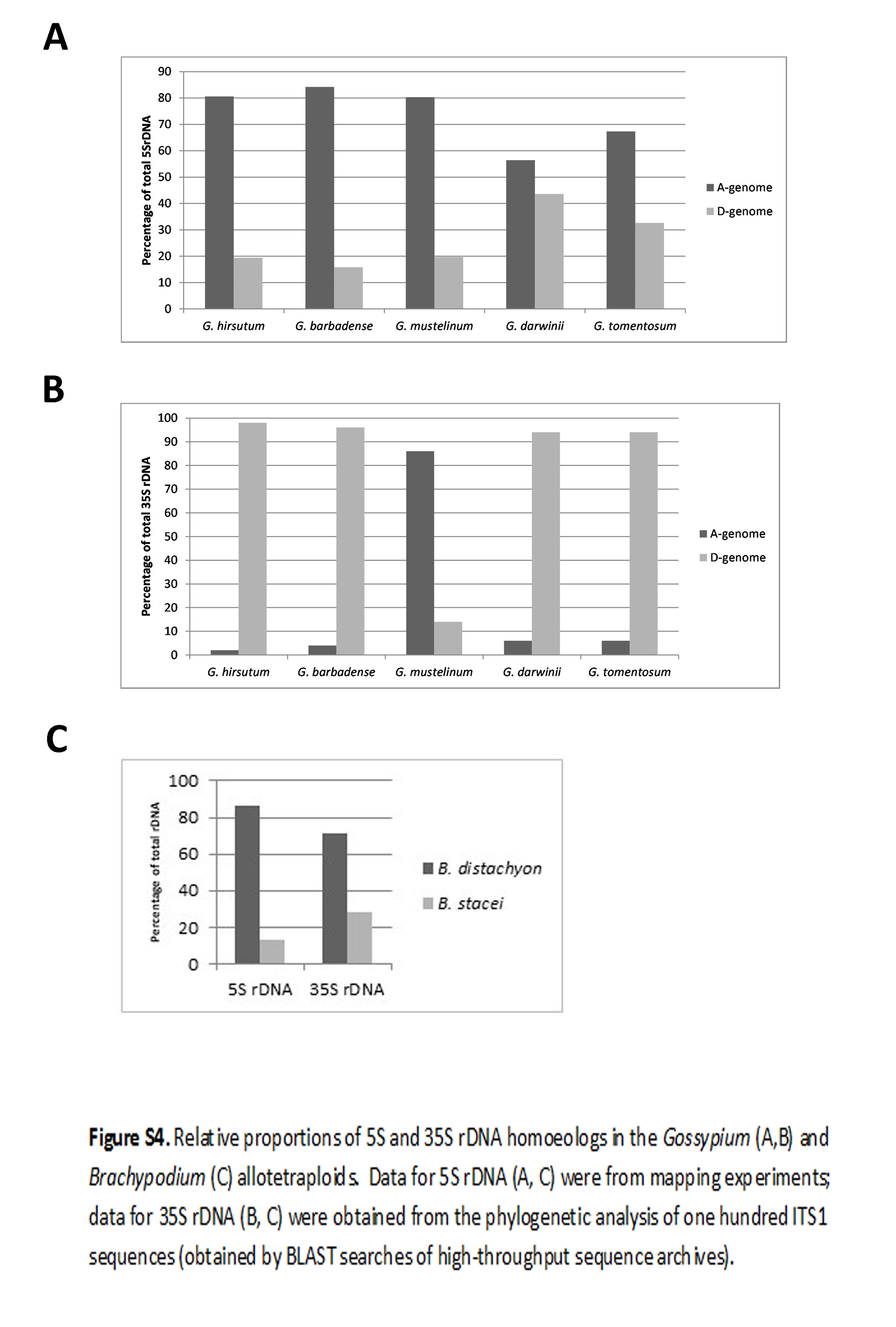

Supplement: Supplementary file 4 [file Image_4.jpeg]

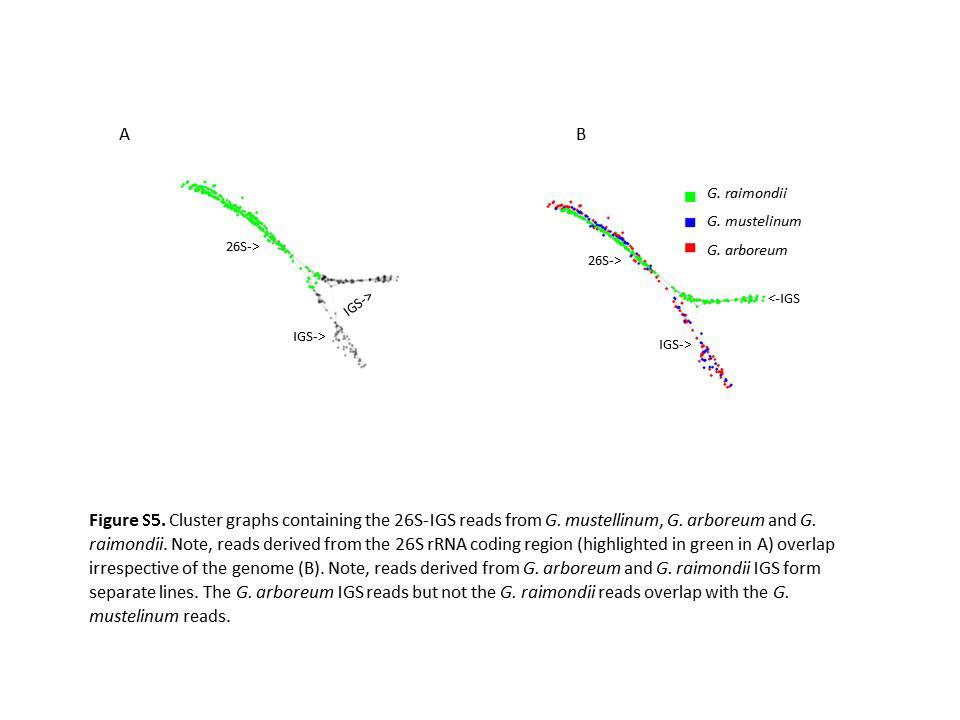

Supplement: Supplementary file 5 [file Image_5.jpeg]
