# Supplementary material for: The Utility of Graph Clustering of 5S Ribosomal DNA Homoeologs in Plant Allopolyploids, Homoploid Hybrids, and Cryptic Introgressants
Source: Front Plant Sci. 2020 Feb 10;11:41. doi: 10.3389/fpls.2020.00041 (PMC7025596; doi:10.3389/fpls.2020.00041)
Supplement: Supplementary file 6 [file Table_1.docx]

**Table S1**. List of species used in the study, ploidy levels, type of graph structures, cluster parameters and sequence read archives accessions.

| Species | **Ploidy level** | **Number of loci/1C^1^** | **Sequence read archive (ID)^2^** | **Connected**  **component index^3^** | **k-mer score^4^** | **Circular cluster type^5^** |
| --- | --- | --- | --- | --- | --- | --- |
| *Arabidopsis arenicola* | 2x | n.d. | ERR2182759 | 0.976 | 0.837 | 1 |
| *Arabidopsis arenosa* | 4x | 3 | SRR2082782 | 0.970 | 0.666 | 2 |
| *Arabidopsis hallerii* | 2x | n.d. | SRR4026781 | n.a.^7^ | n.a. | n.a. |
| *Arabidopsis kamchaticha* | 4x | n.d. | DRR054581 | 0.683 | 0.988 | 2 |
| *Arabidopsis lyrata* | 2x | n.d.^6^ | ERR2182766 | 0.964 | 0.786 | 1 |
| *Arabidopsis suecica* | 2x (4x) | 4 | SRR2084157 | 0.980 | 0.825 | 2 |
| *Arabidopsis thaliana* | 2x | 2 | ERR171440R | 0.995 | 0.960 | 1 |
| *Brachypodium distachyon* | 2x | 1 | SRR3419466 | 0.982 | 0.680 | 1 |
| *Brachypodium hybridum* | 4x | 2 | SRR7068285 | 0.981 | 0.810 | 2 |
| *Brachypodium stacei* | 2x | 1 | SRR3944698 | 1.000 | 0.950 | 1 |
| *Brassica carinata* | 4x | 2 | SRR8126739 | 0.988 | 0.630 | 2 |
| *Brassica napus* | 4x | 4 | ERR424135 | 0.947 | 0.676 | 2 |
| *Brassica nigra* | 2x | 1 | SRR3708162 | 0.989 | 0.904 | 1 |
| *Brassica olearacea* | 2x | 1 | ERR457740 | 0.987 | 0.781 | 1 |
| *Brassica rapa* | 2x | 3 | SRR1296482 | 0.984 | 0.660 | 2 |
| *Capsicum annum* | 2x | 1 | SRR2752023 | 0.984 | 0.686 | 1 |
| *Cardamine amara* | 2x | 1 | this work | 0.979 | 0.830 | 1 |
| *Cardamine flexuosa* | 4x | 1 | this work | 0.956 | 0.722 | 1 |
| *Cardamine hirsuta* | 2x | 1 | this work | 0.989 | 0.946 | 1 |
| *Cardamine insueta* | 3x | n.d. | this work | 0.926 | 0.447 | 2 |
| *Coffea arabica* | 4x | 2 | SRR5602572 | 0.921 | 0.681 | 2 |
| *Cucumis pepo* | 2x | 1 | SRR2531259 | 0.993 | 0.760 | 1 |
| *Ephedra altissima* | 4x | 15 | ERR845262 | 0.993 | 0.629 | 2 |
| *Fritillaria imperialis* | 2x | n.d. | ERR845263 | n.a. | n.a. | 1^8^ |
| *Genlisea nigrocaulis* | 2x | 1 | SRR1049544 | 0.870 | 0.737 | 1 |
| *Glycine dolichocarpa* | 4x | n.d. | SRR1174380 | 0.988 | 0.667 | 2 |
| *Glycine syndetika* | 2x | n.d. | SRR1176843 | 1.000 | 0.846 | 1 |
| *Glycine tomentolla* | 2x | n.d. | SRR1176933 | 0.990 | 0.916 | 1 |
| *Gnetum gnemon* | 2x | n.d. | [ERR268420](https://www.ebi.ac.uk/ena/data/view/ERR268420) | 0.981 | 0.606 | 1 |
| *Gossypium arboreum* | 2x | 1 | SRR1216970 | 1.000 | 1.000 | 1 |
| *Gossypium barbadense* | 4x | 2 | SRR8624709 | 0.923 | 0.581 | 2 |
| *Gossypium darwinii* | 4x | 2 | SRX5347640 | 0.945 | 0.513 | 2 |
| *Gossypium davidsonii* | 2x | 1 | SRR8136261 | 0.991 | 0.738 | 2 |
| *Gossypium gossypioides* | 2x | n.d. | SRR8136267 | 0.852 | 0.713 | 2 |
| *Gossypium herbaceum* | 2x | 1 | SRR617255 | 0.984 | 0.802 | 1 |
| *Gossypium hirsutum* | 4x | 2 | SRR768357 | 0.997 | 0.660 | 2 |
| *Gossypium mustelinum* | 4x | 2 | SRR769542 | 0.990 | 0.624 | 2 |
| *Gossypium raimondii* | 2x | 1 | ERR1449077 | 0.974 | 0.974 | 1 |
| *Gossypium thurberi* | 2x | 1 | SRR8076131 | 1.000 | 0.823 | 1 |
| *Gossypium tomentosum* | 4x | 2 | SRR8815512 | 0.958 | 0.535 | 2 |
| *Chenopodium pallidicaule* | 2x | n.d. | SRR4425239 | 0.684 | 0.988 | 1 |
| *Chenopodium quinoa* | 4x | 2 | DRR057247 | 0.967 | 0.820 | 2 |
| *Chenopodium suecicum* | 2x | n.d. | SRR4425238 | 0.991 | 0.855 | 1 |
| *Musa acuminata ssp.burmannica* | 2x | 4 | SRR7012752 | 0.990 | 0.416 | 2 |
| *Musa balbisiana* | 2x | 3 | SRR6996488 | 0.993 | 0.715 | 2 |
| *Nicotiana benthamiana* | 4x | n.d. | SRR7540368 | 0.987 | 0.680 | 1 |
| *Nicotiana nudicaulis* | 4x | 1 | SRS307170 | 0.984 | 0.927 | 1 |
| *Nicotiana obtusifolia* | 2x | 1 | SRR452993 | 0.932 | 0.596 | 1 |
| *Nicotiana paniculata* | 2x | 1 | SRR8173261 | 0.984 | 0.940 | 1 |
| *Nicotiana repanda* | 4x | 1 | SRR453021 | n.a. | n.a. | n.a. |
| *Nicotiana rustica* | 4x | 2 | SRR8173848 | 0.895 | 0.624 | 2 |
| *Nicotiana sylvestris* | 2x | 1 | SRR343066 | 0.905 | 0.910 | 1 |
| *Nicotiana tabacum* | 4x | 2 | SRR954964 | 0.879 | 0.710 | 2 |
| *Nicotiana tomentosiformis* | 2x | 1 | SRR343065 | 0.862 | 0.962 | 1 |
| *Nicotiana undulata* | 2x | 1 | SRR8173255 | 1.000 | 0.933 | 1 |
| *Prunus avium* | 2x | 1 | SRR9222867 | 0.782 | 0.692 | 1 |
| *Prunus cerasus* | 4x | 2 | SRX5486273 | 0.929 | 0.602 | 2 |
| *Prunus mume* | 2x | 1 | SRR5052852 | 0.998 | 0.938 | 1 |
| *Quercus acuta* | 2x | 1 | ERR2215907 | 0.984 | 0.812 | 1 |
| *Quercus petraea* | 2x | 1 | ERR3284901 | 0.988 | 0.962 | 1 |
| *Quercus robur* | 2x | 1 | SRR2053077 | 0.992 | 0.959 | 1 |
| *Quercus vaseyana* | 2x | 1 | SRR2053036 | 1.000 | 0.959 | 1 |
| *Rosa canina* | 5x | 5 | ERR1662939 | 0.921 | 0.690 | 2 |
| *Rosa corymbifera* | 5x | 5 | SRR8265810 | 0.941 | 0.589 | 2 |
| *Rosa dumalis* | 5x | 6 | ERR1662941 | 0.984 | 0.770 | 2 |
| *Rosa inodora* | 5x | 6 | ERR1662940 | 0.976 | 0.710 | 2 |
| *Rosa majalis* | 2x | n.d. | SRR8422953 | 1.000 | 0.910 | 1 |
| *Rosa moshata* | 2x | n.d. | SRR7077022 | 0.919 | 0.872 | 1 |
| *Rosa multiflora* | 2x | n.d. | [DRR059735](https://www.ebi.ac.uk/ena/data/view/DRR059735) | 0.991 | 0.890 | 1 |
| *Rosa spinosissima* | 4x | n.d. | SRR8422951 | 0.993 | 0.800 | 2 |
| *Secale cereale* | 2x | 1 | ERR505041 | 0.921 | 0.660 | 1 |
| *Senecio vulgaris* | 2x | 1 | SRX1142054 | 0.839 | 0.741 | 1 |
| *Solanum lycopersicum* | 2x | 1 | SRR8205664 | 1.000 | 0.932 | 1 |
| *Spartina alterniflora* | 6x | 1 | this work | 0.976 | 0.870 | 1 |
| *Spartina anglica* | 12x | 2 | this work | 0.833 | 0.58 | 2 |
| *Spartina maritima* | 6x | 1 | this work | 0.943 | 0.628 | 2 |
| *Spartina townsendii* | 6x | 2 | this work | 0.947 | 0.580 | 2 |
| *Spirodela polyrhiza* | 2x | 1 | SRR7548940 | 0.972 | 0.850 | 1 |
| *Theobroma cacao* | 2x | n.d. | SRR3286313 | 1.000 | 0.980 | 1 |
| *Thinopyrum intermedium* | 6x | 3 | SRR5754836 | 0.957 | 0.636 | 2 |
| *Tragopogon dubius* | 2x | 1 | this work | 0.997 | 0.701 | 1 |
| *Tragopogon porrifolius* | 2x | 2 | this work | 0.762 | 0.580 | 2 |
| *Triticum thurgidum* ssp. *durum* | 4x | 2 | SRR567549 | 0.898 | 0.770 | 1 |
| *Zea mays* | 2x | 1 | SRR512996 | 1.000 | 0.940 | 1 |

^1^ Data taken from the database: http://www.plantrdnadatabase.com/ (Garcia et al., 2012)

^2^ The European nucleotide archive (ENA) repository ENA (https://www.ebi.ac.uk/ena) was used to retrieve sequences

^3^  Reported by RepeatExplorer in “Cluster annotation“ or “TAREAN“ windows

^4^  Reported by RepeatExplorer in “Cluster annotation>report“ Windows

^5^ Defined based on **Figure 1**.

^6.^ n.d. – not determined

^7^n.a. – not applicable, no TAREAN record was obtained

^8^ Graph type determined by visual inspection only.
