# Supplementary material for: The Utility of Graph Clustering of 5S Ribosomal DNA Homoeologs in Plant Allopolyploids, Homoploid Hybrids, and Cryptic Introgressants
Source: Front Plant Sci. 2020 Feb 10;11:41. doi: 10.3389/fpls.2020.00041 (PMC7025596; doi:10.3389/fpls.2020.00041)
Supplement: Supplementary file 7 [file Table_2.docx]

| **Table S2 - Statistical evaluation of the relationships between cluster shape, ploidy level and locus number** | | | | |
| --- | --- | --- | --- | --- |
|  |  |  |  |  |
| **Number of species showing Type 1 and Type 2 graph structures** | | |  |  |
|  | **Diploids** | **Polyploids** | **Single locus species** | **Multiple loci** |
| **Type 1** | 43 | 5 | 33 | 2 |
| **Type 2** | 7 | 32 | 2 | 27 |
|  |  |  |  |  |
| **Annova one-way comparative test** | | **F** | **P** |  |
| Diploid versus polyploid species | | 75.50678764 | **3.58179E-13** |  |
| Single versus multiple loci species | | 200.363425 | **4.35111E-21** |  |
|  |  |  |  |  |
| **Data sets** |  |  |  |  |
|  |  |  |  |  |
| **Diploids** | **Cluster shape** | **Polyploids** | **Cluster shape** |  |
| *Arabidopsis arenosa* | 2 | *Arabidopsis suecica* | 2 |  |
| *Arabidopsis thaliana* | 1 | *Arabidopsis kamchaticha* | 2 |  |
| *Arabidopsis lyrata* | 1 | *Brachypodium hybridum* | 2 |  |
| *Arabidopsis arenicola* | 1 | *Brassica carinata* | 2 |  |
| *Brachypodium distachyon* | 1 | *Brassica napus* | 2 |  |
| *Brachypodium stacei* | 1 | *Cardamine flexuosa* | 1 |  |
| *Brassica nigra* | 1 | *Cardamine insueta* | 2 |  |
| *Brassica olearacea* | 1 | *Coffea arabica* | 2 |  |
| *Brassica rapa* | 2 | *Ephedra altissima* | 2 |  |
| *Capsicum annum* | 1 | *Glycine dolichocarpa* | 2 |  |
| *Cardamine amara* | 1 | *Gossypium barbadense* | 2 |  |
| *Cardamine hirsuta* | 1 | *Gossypium darwinii* | 2 |  |
| *Cucumis pepo* | 1 | *Gossypium hirsutum* | 2 |  |
| *Fritillaria imperialis* | 1 | *Gossypium mustelinum* | 2 |  |
| *Genlisea nigrocaulis* | 1 | *Gossypium tomentosum* | 2 |  |
| *Glycine syndetika* | 1 | *Chenopodium quinoa* | 2 |  |
| *Glycine tomentolla* | 1 | *Nicotiana rustica* | 2 |  |
| *Gnetum gnemon* | 1 | *Nicotiana nudicaulis* | 1 |  |
| *Gossypium raimondii* | 1 | *Nicotiana tabacum* | 2 |  |
| *Gossypium arboreum* | 1 | *Nicotiana benthamiana* | 1 |  |
| *Gossypium davidsonii* | 2 | *Prunus cerasus* | 2 |  |
| *Gossypium gossypioides* | 2 | *Rosa inodora* | 2 |  |
| *Gossypium herbaceum* | 1 | *Rosa canina* | 2 |  |
| *Gossypium thurberi* | 1 | *Rosa corymbifera* | 2 |  |
| *Chenopodium suecicum* | 1 | *Rosa spinosissima* | 2 |  |
| *Chenopodium palidicaule* | 1 | *Rosa dumalis* | 2 |  |
| *Musa acuminata ssp.burmannica* | 2 | *Spartina alterniflora* | 1 |  |
| *Musa balbisiana* | 2 | *Spartina anglica* | 2 |  |
| *Nicotiana paniculata* | 1 | *Spartina maritima* | 2 |  |
| *Nicotiana undulata* | 1 | *Spartina townsendii* | 2 |  |
| *Nicotiana tomentosiformis* | 1 | *Triticum thurgidum* ssp. *Durum* | 1 |  |
| *Nicotiana sylvestris* | 1 | *Thinopyrum intermedium* | 2 |  |
| *Nicotiana obtusifolia* | 1 |  |  |  |
| *Prunus avium* | 1 |  |  |  |
| *Prunus mume* | 1 |  |  |  |
| *Quercus robur* | 1 |  |  |  |
| *Quercus vaseyana* | 1 |  |  |  |
| *Quercus acuta* | 1 |  |  |  |
| *Quercus petraea* | 1 |  |  |  |
| *Rosa multiflora* | 1 |  |  |  |
| *Rosa majalis* | 1 |  |  |  |
| *Rosa moshata* | 1 |  |  |  |
| *Secale cereale* | 1 |  |  |  |
| *Senecio vulgaris* | 1 |  |  |  |
| *Solanum lycopersicum* | 1 |  |  |  |
| *Spirodela polyrhiza* | 1 |  |  |  |
| *Theobroma cacao* | 1 |  |  |  |
| *Tragopogon dubius* | 1 |  |  |  |
| *Tragopogon porrifolius* | 2 |  |  |  |
| *Zea mays* | 1 |  |  |  |
|  |  |  |  |  |
|  |  |  |  |  |
| **Single locus species** | **Cluster shape** | **Multiple loci species** | **Cluster shape** |  |
| *Brachypodium distachyon* | 1 | *Arabidopsis arenosa* | 2 |  |
| *Brachypodium stacei* | 1 | *Arabidopsis suecica* | 2 |  |
| *Brassica nigra* | 1 | *Arabidopsis thaliana* | 1 |  |
| *Brassica olearacea* | 1 | *Brachypodium hybridum* | 2 |  |
| *Capsicum annum* | 1 | *Brassica carinata* | 2 |  |
| *Cardamine amara* | 1 | *Brassica napus* | 2 |  |
| *Cardamine flexuosa* | 1 | *Brassica rapa* | 2 |  |
| *Cardamine hirsuta* | 1 | *Coffea arabica* | 2 |  |
| *Cucumis pepo* | 1 | *Ephedra altissima* | 2 |  |
| *Genlisea nigrocaulis* | 1 | *Gossypium barbadense* | 2 |  |
| *Gossypium raimondii* | 1 | *Gossypium darwinii* | 2 |  |
| *Gossypium arboreum* | 1 | *Gossypium hirsutum* | 2 |  |
| *Gossypium davidsonii* | 2 | *Gossypium mustelinum* | 2 |  |
| *Gossypium herbaceum* | 1 | *Gossypium tomentosum* | 2 |  |
| *Gossypium thurberi* | 1 | *Chenopodium quinoa* | 2 |  |
| *Nicotiana paniculata* | 1 | *Musa acuminata ssp.burmannica* | 2 |  |
| *Nicotiana undulata* | 1 | *Musa balbisiana* | 2 |  |
| *Nicotiana tomentosiformis* | 1 | *Nicotiana rustica* | 2 |  |
| *Nicotiana nudicaulis* | 1 | *Nicotiana tabacum* | 2 |  |
| *Nicotiana sylvestris* | 1 | *Prunus cerasus* | 2 |  |
| *Nicotiana obtusifolia* | 1 | *Rosa inodora* | 2 |  |
| *Prunus avium* | 1 | *Rosa canina* | 2 |  |
| *Prunus mume* | 1 | *Rosa corymbifera* | 2 |  |
| *Quercus robur* | 1 | *Rosa dumalis* | 2 |  |
| *Quercus vaseyana* | 1 | *Spartina anglica* | 2 |  |
| *Quercus acuta* | 1 | *Spartina townsendii* | 2 |  |
| *Quercus petraea* | 1 | *Tragopogon porrifolius* | 2 |  |
| *Secale cereale* | 1 | *Triticum thurgidum* ssp. *Durum* | 1 |  |
| *Senecio vulgaris* | 1 | *Thinopyrum intermedium* | 2 |  |
| *Solanum lycopersicum* | 1 |  |  |  |
| *Spartina alterniflora* | 1 |  |  |  |
| *Spartina maritima* | 2 |  |  |  |
| *Spirodela polyrhiza* | 1 |  |  |  |
| *Tragopogon dubius* | 1 |  |  |  |
| *Zea mays* | 1 |  |  |  |
|  |  |  |  |  |
|  |  |  |  |  |
