# Supplementary material for: The Utility of Graph Clustering of 5S Ribosomal DNA Homoeologs in Plant Allopolyploids, Homoploid Hybrids, and Cryptic Introgressants
Source: Front Plant Sci. 2020 Feb 10;11:41. doi: 10.3389/fpls.2020.00041 (PMC7025596; doi:10.3389/fpls.2020.00041)
Supplement: Supplementary file 8 [file Table_3.docx]

**Table S3.** Details of genome skimming projects carried out within this study.

| Species | ID/  sampling | Sequencing platform/  Company^1^ | No. of pair end reads^2^ | Read length (bp) | Genome coverage^3^ | GenBank accession No. |
| --- | --- | --- | --- | --- | --- | --- |
| *Spartina alterniflora* | 2017 collection, Hythe, UK | Illumina Hiseq4000/ BGI | 17,477,504 | 150 | 1.23 | SRR10224435 |
| *Spartina maritima* | 2017 collection, site 2, Isle of Wight, UK | Illumina Hiseq4000/ BGI | 17,481,886 | 150 | 1.39 | SRR10224434 |
| *Spartina* × *townsendii* | 2016 collection, M8 site  Ealing Marchwood, UK | Illumina Hiseq4000/ BGI | 16,286,542 | 150 | 1.21 | SRR10224433 |
| *Spartina anglica* | 2016 collection, M5 site,  Ealing Marchwood, UK | Illumina Hiseq4000/ BGI | 17,428,486 | 150 | 0.49 | SRR10224432 |
| *Cardamine amara* | Urnerboden, Switzerland | Illumina Hiseq4000/ BGI | 18,759,630 | 150 | 11.82 | SRR10230724 |
| *Cardamine* × *insueta* | Urnerboden, Switzerland | Illumina Hiseq2500/Eurofins Genomics | 40,590,158 | 125 | 2.69 | SRR10230723 |
| *Cardamine flexuosa* | Železné,  Slovakia | Illumina Hiseq2500/Eurofins Genomics | 30,306,672 | 125 | 4.30 | SRR10230721 |
| *Cardamine hirsuta* | Gehausen, Germany ( Oxford accession,  UK) | Illumina Hiseq2500/Eurofins Genomics | 38,279,056 | 125 | 21.36 | SRR10230722 |

^1^ Small fragment library, PCR free.

^2^ Total number of reads after adaptor trimming and low quality reads removal.

^3^ Genome coverage was calculated as the number reads*read length/genome size. Genome size data were taken from the Plant DNA C-values Database (Leitch et al., 2019). The genome size of the 6x *S.* × *townsendii* hybrid was calculated as the mean of *the S. alterniflora* and *S. maritima* parents. The data from *C.* × *insueta* are taken from (Mandakova et al., 2013).

References

Leitch, I.J., Johnston, E., Pellicer, J., Hidalgo, O., and Bennett, M.D. (2019). Plant DNA C-values database (release 7.1, Apr 2019) <https://cvalues.science.kew.org/> [*http://data.kew.org/cvalues/*](http://data.kew.org/cvalues/)*.*

Mandakova, T., Kovarik, A., Zozomova-Lihova, J., Shimizu-Inatsugi, R., Shimizu, K.K., Mummenhoff, K., et al. (2013). The More the Merrier: Recent hybridization and polyploidy in *Cardamine*. *Plant Cell* 25**,** 3280-3295. doi: 10.1105/tpc.113.114405.
